# Cerebellar Information Processing in Relapsing-Remitting Multiple Sclerosis (RRMS)

**DOI:** 10.3233/BEN-2010-0267

**Published:** 2010-08-16

**Authors:** E. Lesage, M. A. J. Apps, A. L. Hayter, C. F. Beckmann, D. Barnes, D. W. Langdon, N. Ramnani

**Affiliations:** ^1^Department of PsychologyRoyal HollowayUniversity of LondonLondonUK; ^2^Division of Neuroscience and Mental HealthImperial College LondonLondonUK; ^3^Centre for Functional MRI of the BrainUniversity of OxfordOxfordUK; ^4^Ashford and St Peter’s HospitalUK

## Abstract

Recent research has characterized the anatomical connectivity of the cortico-cerebellar system – a large and important fibre system in the primate brain. Within this system, there are reciprocal projections between the prefrontal cortex and Crus II of the cerebellar cortex, which both play important roles in the acquisition and execution of cognitive skills. Here, we propose that this system also plays a particular role in sustaining skilled cognitive performance in patients with Relapsing-Remitting Multiple Sclerosis (RRMS), in whom advancing neuropathology causes increasingly inefficient information processing. We scanned RRMS patients and closely matched healthy subjects while they performed the Paced Auditory Serial Addition Test (PASAT), a demanding test of information processing speed, and a control task. This enabled us to localize differences between conditions that change as a function of group (group-by-condition interactions). Hemodynamic activity in some patient populations with CNS pathology are not well understood and may be atypical, so we avoided analysis strategies that rely exclusively on models of hemodynamic activity derived from the healthy brain, using instead an approach that combined a ‘model-free’ analysis technique (Tensor Independent Component Analysis, TICA) that was relatively free of such assumptions, with a post-hoc ‘model-based’ approach (General Linear Model, GLM). Our results showed group-by-condition interactions in cerebellar cortical Crus II. We suggest that this area may have in role maintaining performance in working memory tasks by compensating for inefficient data transfer associated with white matter lesions in MS.

## 1. Introduction

Relapsing-Remitting Multiple Sclerosis (RRMS) is the most common form of multiple sclerosis, a degenerative Central Nervous System (CNS) condition characterized by inflammation and demyelination [28]. The disease pattern is characterized by alternating periods of relapse where patients exhibit symptoms, and phases in which patients are largely asymptomatic. During these phases of remission, performance on motor and cognitive tests is within, or close to, the normal range. Thus, the CNS must in some way compensate for impaired information processing caused by white matter degeneration and other pathology [43,71]. While some models of compensation emphasize local, perilesional reorganisation, others emphasize large-scale systems level reorganisation. Functional neuroimaging has been used to study these large scale changes in patients in whom pathology is relatively localized (such as in stroke [41,84], or relatively diffuse (such as in Alzheimer disease [65]. It is likely that since the distribution of CNS pathology differs to some extent across patients, the consequent pattern of reorganisation is likely to be unique in each patient. However, functional

ISSN 0953-4180/10/$27.50 © 2010 – IOS Press and the authors. All rights reserved neuroimaging studies have revealed that there are patterns of reorganisation that are common across a given patient population [69].

It has been previously suggested that the plastic properties of some neural systems that allow them to support learning might also allow them to support the recovery of function during disease progression or acute injury [58]. The cortico-cerebellar system is well known for its contributions to the acquisition of motor and cognitive skills [55,77]. The cerebellar cortex operates in concert with cortical motor and prefrontal areas to support motor and cognitive skills [4] and there is known to exist anatomical and functional topography within the cerebellar cortex that distinguishes its interactions with the cortical motor system and with the prefrontal cortex [77]. It has previously been suggested that areas of the cerebellar cortex that are interconnected with the prefrontal cortex (medial and hemispheral portions of lobule VII, including Crus I and Crus II [56]) may support the acquisition and execution of cognitive skills, making this area a candidate structure for supporting cognitive skills in RRMS patients. Indeed, RRMS patients with clinical cerebellar dysfunction (ataxic gait and nystagmus) demonstrated more cognitive dysfunction than RRMS patients without cerebellar signs [88]. The cerebellar cortex is particularly vulnerable to demyelination in MS (although this is less pronounced in RRMS) and this is often independent of white matter lesions [61]. It is likely that grey matter demyelination and other pathology besides white matter lesions will emerge as equally important influences on cerebellar function [25]. However these phenomena remain to be demonstrated as yet in the relapsing remitting subtype [6].

The PASAT [46] is a widely used test of information processing speed. The auditory processing and verbal working memory demands of this test (see ‘Methods’) include the requirement to coordinate several cognitive processes. The PASAT is particularly sensitive to reduced cognitive abilities in MS patients [78,79], however, during the remitting phases of RRMS, some patients perform the PASAT at the same level of competence as matched healthy controls, suggesting the acquisition of cognitive skills. Previous functional neuroimaging studies have reported that areas of the cerebellar cortex are activated by the demands of the PASAT in healthy controls [23,42]. In a previous study, Hayter et al. [50] developed a variant of the PASAT which could be used in its original form during fMRI using a ‘sparse sampling’ technique (see Methods). This study showed PASAT-related activity in bilateral parts of the prefrontal cortex as well as bilaterally in medial parts of lobule VII in young healthy subjects.

Here, we investigate whether Crus II plays a specific role in supporting cognitive performance in RRMS using fMRI. Our experimental design allowed us to test for a ‘Group-by-Condition’ interaction in which the differences between PASAT-related activity and a control condition were greater in a group of RRMS patients relative to group of closely matched healthy control participants. We also make use of the advantages offered by Independent Component Analysis, which, unlike conventional analysis techniques, does not rely on the accuracy of assumptions about the temporal dynamics of the BOLD signal in individual subjects.

## 2. Methods

Our experimental design and fMRI data acquisition methods replicate those reported in Hayter et al. [50].

### 2.1. Participants

Initially, twenty-two right-handed patients diagnosed with clinically definite Relapsing Remitting Multiple Sclerosis [74–76] and a matched control group, consisting of normal healthy volunteers, were recruited. Participants were excluded from both groups if they were aged over 60 years, had a history of chronic medical disorders (other than MS for the patient group), alcohol or drug abuse, psychiatric disorders, head injury resulting in loss of consciousness, or any contraindications that would prohibit MRI scanning. MS patients were at least one month post-relapse. Participants were required to perform the PASAT with the stimuli numbers spoken every three seconds (PASAT 3 s), at a level that was within two standard deviations of a healthy control group mean [22]. Three recruited patients did not meet this criterion and were subsequently excluded from the study.

19 RRMS patients (15 females; mean (M) age 41.7 years; age range 29–55) comprised the patient group. The mean time since diagnosis was 6.4 years. The mean disability score was 2.7 on the Hauser Ambulatory Index (HAI; [49]). Fourteen patients were on Disease Modifying Therapy (DMT). The Control group was appropriately matched for handedness, age, gender and premorbid IQ (15 females; mean age 39.1 years, range 26–55).

The study was conducted in accordance with the permission of NHS South West Surrey Ethics committee, the medical devices agency and the Royal Holloway, University of London, Psychology Department Ethics Committee. All participants gave written informed consent.

### *2.2. Apparatus*

Participants lay supine in the scanner. Sound was delivered through MRI-compatible headphones (see [50]). Speech was recorded using a custom built MRI-compatible microphone. Visual instructions were presented on a screen which subjects observed using an overhead mirror mounted on the head coil. Presentation software (Neurobehavioral Systems, Inc., USA) was used to deliver stimuli and synchronize the experiment with the scanner. An A/D 1401 unit (Cambridge Electronic Design, UK) was used to record all stimuli, auditory response waveforms and scanner timings (1kHz or higher) in conjunction with Spike2 software. PASAT performance was scored offline.

### *2.3. Procedure*

There were four phases:

- Phase 1 – Neuropsychological testing to screen for cognitive dysfunction.
- Phase 2 – Immediately prior to scanning, before entering the scanner, participants practiced the PASAT three times and completed an anxiety scale.
- Phase 3 – Once within the scanner, participants practiced the experimental and control tasks (see below), and then completed the main experiment with the same conditions once they were proficient.
- Phase 4 – T1 and T2 FLAIR Anatomical image acquisition (see Image Acquisition below).

#### *2.3.1. Phase 1: Neuropsychological Testing Procedures*

Premorbid IQ (Wechsler Test of Adult Reading WTAR [90]) and current IQ (pro-rated from the Wechsler Abbreviated Scale of Intelligence, WASI, Vocabulary and Similarities subtests [90]) were measured. Cognitive functioning was further assessed using the VESPAR (Verbal and Spatial Reasoning Test, Verbal and Spatial Analogy Subtests [63]) and the BRB-N (Brief Repeatable Battery of Neuropsychological Tests in Multiple Sclerosis [20]). The BRB-N comprises five tests. The most sensitive to MS are the PASAT (with the numbers spoken first every three seconds, PASAT 3 s, and then at the faster rate of every two seconds, PASAT 2 s) and the Symbol Digit Modality Task (SDMT, a coding task with spoken number responses [85]). Manual dexterity (9-hole peg test [29]), affect (Hospital Anxiety and Depression Scale, HADS [92]) and fatigue (Fatigue Severity Scale, FSS [60]) were also assessed. Increased prevalence of depression and anxiety [21] as well as fatigue [59] has been documented in MS. Differences between patients and controls on these cognitive tasks were examined using 2-sample t-tests. In order to assess whether these measures related to performance, correlations were analyzed between the cognitive tests and anxiety, depression and fatigue scores using Pearson's correlation coefficient.

#### *2.3.2. Phase 2: PASAT practice*

The remaining phases were all completed on one day, within two months of the neuropsychological battery. To ensure that PASAT performance reached stable asymptotic levels prior to scanning [13,87], both patients and control participants underwent three training sessions of the 3 second PASAT immediately before fMRI scanning. State anxiety was also measured (Speilberger State Trait Anxiety Inventory, STAI short form [67]).

#### *2.3.3. Phase 3: fMRI Experiment*

The experimental design and imaging methodology was adapted from a previously published study [50]. The study followed a $2 \times 2$ factorial experimental design: Factor 1 was Group (patients vs. controls) and factor 2 was Condition (experimental vs. control). This design thus enabled us to test for Group-by-Condition interactions (differential activity between the levels of Condition that was different in each level of Group). The study employed a block-design. Experimental and control blocks were pseudo-randomly ordered. Each block consisted of 5 single digits, presented aurally, one every 3 seconds. Every participant performed both conditions, constituting a repeated measures design. In the experimental condition (ADD), participants were required to add each digit to the immediately preceding one and speak their response (akin to the PASAT). In the control condition (REPEAT), participants were also required to make a verbal response, but they were only required to repeat each digit after hearing it. Hence, sensory and motor demands were matched to those in the experimental condition. In the period between blocks, a visual instruction cue was presented that indicated which of the two conditions was about to follow. The words "ADD" or "REPEAT" were presented in black text against a grey background for 1000ms (see 'Experimental Timings' below).

During the practice session within the scanner (prior to the main experiment) participants were presented with 2 blocks of trials for each condition, under circumstances identical to those of the following experiment. This allowed them to become familiar with the specific context of performing both conditions during scanning. During the main experiment which followed, participants were presented with 35 blocks of each condition and 10 null blocks in which no stimuli were presented (duration of main experiment: 32 minutes). Conditions were pseudo-randomly intermixed.

#### *2.3.4. Phase 4: Acquisition of anatomical images*

(see Image Acquisition below).

### *2.4. Experimental timings: Block lengths and sparse sampling*

These methods are replicated from Hayter et al. [50]. Each block lasted for 15 seconds (5 TRs), and was followed by a rest period of 9 seconds (3 TRs) before the onset of the following block. Instruction cues were jittered uniformly and randomly over the 9 second rest period between blocks. Tasks that require subjects to make verbal responses are likely to incur head motion-related artifacts in EPI images [17–19]. To overcome this problem, a 'sparse sampling' technique was employed. A period of scanner silence was introduced at the time that verbal responses are produced by participants (no EPI images are collected in this period; this is also known as compressed block design or clustered volume acquisition [39,40,47,50]. This method takes advantage of the slow time course of the BOLD response, such that scans can capture the hemodynamic outcomes of information processing long after the neural processes that cause them have ended. Although originally designed to take advantage of the silent period for delivery of sounds [7,8,10,11,16,34,47,70], this method has also been shown to minimize head-motion artifacts in speech production tasks [1,39,45,50]. In this study head motion parameters were checked to ensure that this was the case. The following exclusion criteria were set to realignment parameters; rotations were not allowed to exceed 2° and translations could not exceed 3mm. All participants met this requirement. Within each TR (3 s) 27 slices were acquired in 2 s, leaving a 1 s silent period.

### *2.5. Image Acquisition*

Participants were scanned using a 3 Tesla Siemens Trio MRI scanner (http://www.pc.rhul.ac.uk/web/Research/mri.htm). In Phase 3, 644 EPI images were acquired (transversal orientation, TE = 32 ms; TR = 3 s; 27 slices; 4 mm thickness; in plane voxel dimension = 3 mm$^2$; flip angle = 90o; FoV = 192 $\times$ 192; image matrix = 64 $\times$ 64 pixels). The experiment began after 4 volumes were collected to minimize T1 relaxation artifacts. For Phase 4, structural images were acquired from both patients and controls using a T1 MPRAGE sequence (TE = 5.56 ms; TR = 1830 ms; T1 = 1100 ms, voxel size 1 $\times$ 1 $\times$ 1 mm, slices = 160). These were used to assist with anatomical localisation of activations. A T2 weighted 2D FLAIR sequence was obtained for patients only (TE = 118 ms; TR = 7500 ms; TI = 3000 ms; voxel size 1 $\times$ 1 $\times$ 3 mm, slices = 44) for visualizing lesions.

### *2.6. Data analysis*

Our analysis strategy combines the advantages of model-based and model-free techniques. Independent Component Analysis (ICA) is a blind-source separation technique that extracts independent non-Gaussian components from multivariate signals. The advantage of this approach is that it requires few a priori assumptions about the exact temporal characteristics of signals. For example, studies of hemodynamic response properties have shown that BOLD responses vary significantly between subjects as well as between brain regions [2,48]. Moreover, systematic differences between the hemodynamic response properties in healthy subjects and in elderly [35] and patient populations [24] have been reported. The use of ICA is therefore a particular advantage in clinical populations in which neurovascular coupling (and therefore hemodynamic response profiles) is not well understood. One potential disadvantage of this analysis is that the resulting components are not easily interpreted. Our approach therefore applies a post-hoc model-based linear regression to the outputs of ICA, such that we were able to identify components of which the timecourses explain the experimental variance maximally (see below).

Multi-Subject analysis was carried out using Tensorial Independent Component Analysis (TICA [15]) as implemented in MELODIC (Multivariate Exploratory Linear Decomposition into Independent Components) Version 3.09, within FSL (FMRIB's Software Library, www.fmrib.ox.ac.uk/fsl).

The data was preprocessed by masking non-brain voxels, voxel-wise de-meaning of the data and normalisation of the voxel-wise variance (see [14] for details). All preprocessed data sets were whitened and projected into a 75-dimensional subspace using Principal Component Analysis. The whitened observations from all subjects were simultaneously decomposed into sets of vectors which describe signal variation across the temporal domain (time-courses), the session/subject domain and across the spatial domain (maps) by optimising for non-Gaussian spatial source distributions using a fixed-point iteration technique [51]. Every estimated component consists of a spatial map which indicates locality of signal, a single time course which represents the temporal dynamics (on average across the population) of an underlying signal and a third vector which describes the subject-specific effect size of the signal represented within the component.

We can test for statistical significance of each one of these vectors. Firstly, the spatial component maps were divided by the standard deviation of the residual noise and thresholded by fitting a Gaussian mixture model to the histogram of intensity values [14] in order to identify voxels which show significant effect size on average. Secondly, every components time course was tested against explanatory variables that model the instruction cue and the ADD and REPEAT conditions within the framework of a standard General Linear Model (GLM). Thirdly, the subject-specific effect size estimates were tested within a GLM against a predictor variable of Group membership (RRMS patients vs. matched controls).

We selected a component for further analysis based two criteria that indicate a task-related pattern of activation. First, we examined the spectral properties of the timecourse. To ensure that the component revealed task-related activations, the frequency of the component had to match the frequency of experimental events. The second criterion was the outcome of the post-hoc regression analysis. We selected the component that maximally distinguished between ADD and REPEAT conditions. The reported results focus on the component that best conformed to these criteria.

Subject-specific average timecourses were extracted from clusters of interest. For every experimental block, timecourses within blocks were normalized with respect to the BOLD amplitude one at the TR before block onset. We then approximated the area under the block-related hemodynamic response curve by taking the sum of the BOLD amplitudes for each TR in the peristimulus interval. In order to verify the presence of main effects and interactions in the clusters, a mixed model analysis with Condition and Group as fixed effects and Block and Subject as random effects was then conducted using SPSS software. These analyses enabled us to make statistical inferences about the activity in specific regions within the selected component.

### 2.7. *Anatomical localization*

The atlas of Duvernoy and Bourgouin [38] was used as a general neuroanatomical reference. We examined the location of the cerebellar cluster with the cerebellar atlas by Diedrichsen, Balsters, Flavell, Cussans, and Ramnani [36]. The nomenclature of Larsell and Jansen [64] was used to label cerebellar regions. Subject-specific clusters were superimposed on the participants' anatomical images to verify that activations were in the same anatomical regions in every participant.

## 3. Results

### 3.1. *Neuropsychological Battery and FLAIR Lesion Load Results*

The matching process ensured there were no significant differences in age, gender, estimated premorbid and current IQ between the groups. Patients exhibited no significant difference between premorbid and current IQ scores, indicating that there was no global intellectual deterioration. The MS patients did not significantly differ from control participants on any cognitive test, except for the SDMT, where the control participants scored significantly higher than the MS patients (t(36) $= -2.3$, $p = 0.04$). When cut-off scores from the healthy control group were applied (i.e. below 2 standard deviations from the mean), only three patients were designated impaired on the SDMT, whereas all other tests were performed within normal limits by every patient. Comparison of the group means on both the PASAT 3 s and the PASAT 2 s showed no statistical difference.

A significant difference was found between groups on anxiety, depression (HADS [92]) and fatigue scores (FSS ci59,60). Patients had significantly higher ratings for trait anxiety, depression and fatigue compared to controls. However, these factors did not correlate significantly with patients' performance on the first exposure to the PASAT 3 seconds (Pearson's correlation coefficient, $p > 0.05$, two-tailed). There were no signif-

Table 1
Participants descriptive statistics and independent t-tests on PASAT tests, at neuropsychological testing, pre-fMRI practice, and during fMRI

| Test | MS patients mean (SD) | Control participants mean (SD) |
|---|---|---|
| Practice 1 (PASAT 3 seconds) | 48.3(7.4) | 52.1(5.6) |
| Practice 2 | 52.0(6.2) | 54.2(4.8) |
| Practice 3 | 51.3(5.8) | 54.9(4.9) |
| fMRI total | 133.6(4.6) | 134.1(3.5) |
| fMRI blocks | 30.2(3.1) | 30.1(2.8) |

icant differences in state anxiety between patients and control participants immediately before scanning.

T2-weighted images are commonly used to reveal white matter pathology and to quantify the structural abnormalities in MS patients (lesion load). The T2 FLAIR images revealed a mean T2 lesion load of 20.9 $mm^3$ (SD = 21.5 $mm^3$). The T2 lesion load did not correlate with any cognitive test for the patient group (Pearson's correlation coefficient, $p > 0.05$, two-tailed), as in Valentino et al. [88]. There was a significant positive correlation between the left ($r = 0.774$, $n = 19$, $p < 0.001$, two-tailed) and right hand ($r = 0.533$, $n = 19$; $p = 0.019$, two-tailed) 9 hole peg test and lesion load.

### 3.2. *PASAT behavioural results*

There were no significant group differences between mean scores at each practice trial of the 3-second PASAT. Both groups showed no significant increase in performance level between the last two practices (third and fourth exposures), indicating that both groups were fully practiced by the time the fMRI experiment began.

In Phase 3, during the experimental task, patients ($M = 133.6$, SD = 4.6) and controls ($M = 134.1$, SD = 3.5) showed similar performance on the PASAT task (maximum score 145) within the scanner (Table 1), yielding no significant difference (t(36) = −0.4, $p > 0.05$).

### 3.3. *Imaging results*

In order to conserve processing resources, MELODIC was set to extract up to 75 components from the data on the basis of their independence from each other and departure from a Gaussian distribution. Voxels within a given component have similar temporal characteristics. We selected the component which had the highest Z value for the post-hoc GLM contrast for ADD > REPEAT ($z = 26.8$; $p < 0.001$), and also had a timecourse that cycled at the fundamental frequency of the experiment (blocks were 24 s between – we found a spectral peak around this fundamental frequency; 0.0416 Hz (1/24 s); see Fig. 1). It is important to note that this frequency relates to a contribution from both conditions. As one would expect from our experimental design, there are also smaller peaks at lower frequencies. These likely reflect the periodicity of ADD and REPEAT blocks.

The selected component contained a large cluster within cerebellar cortical Crus II (peak MNI coordinate: 34, −59, −40; $Z = 3.08$). Timecourses from this cluster were extracted and further analysed to verify the presence of main and interaction effects of Group and Condition.

A mixed-effects analysis and examination of the peri-stimulus time plot derived from the average timecourse from the cluster identified in the cerebellar cortex showed a clear Group-by-Condition interaction, where the effect of ADD > REPEAT was larger in the patient group than in the control group (F(1, 32.822) = 10.277, $p = 0.009$; see Fig. 3). This result demonstrates that the cerebellar cortex responds more robustly to the cognitive demands of the PASAT in RRMS patients than in matched controls, despite the fact that the groups did not significantly differ in their performance. We suggest that this is consistent with the view that cerebellar cortical areas that are interconnected with the prefrontal cortex support compensatory processes that assist the execution of acquired cognitive skills in RRMS patients.

## 4. Discussion

The aim of our study was to investigate neural activity related to compensatory processes in RRMS patients during the skilled execution of the PASAT – a demanding test of information processing speed that is sensitive to MS pathology. Our approach was to determine differential activations in RRMS patients and matched controls, whilst matching cognitive performance during scanning. Thus, performance differences could not explain the results. Our results consistent that while areas in the prefrontal cortex operate similarly in the two groups, there are significant differences between groups in the areas of the cerebellar cortex that exchange information with these prefrontal areas. Our results are consistent with the view that the cerebellar cortex contributes importantly to the reorganization of information flow to maintain cognitive performance in the face of white matter degeneration and other cere-

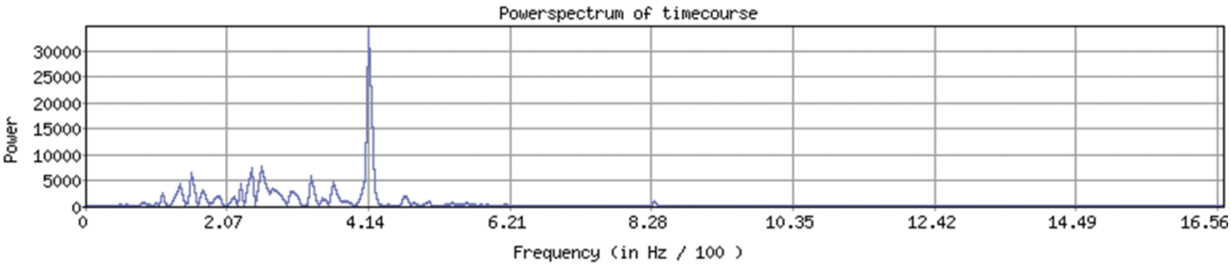

Fig. 1. Spectral properties for the selected component. The timecourse of this component has a frequency matching the frequency of the experiment (1/inter-block interval).

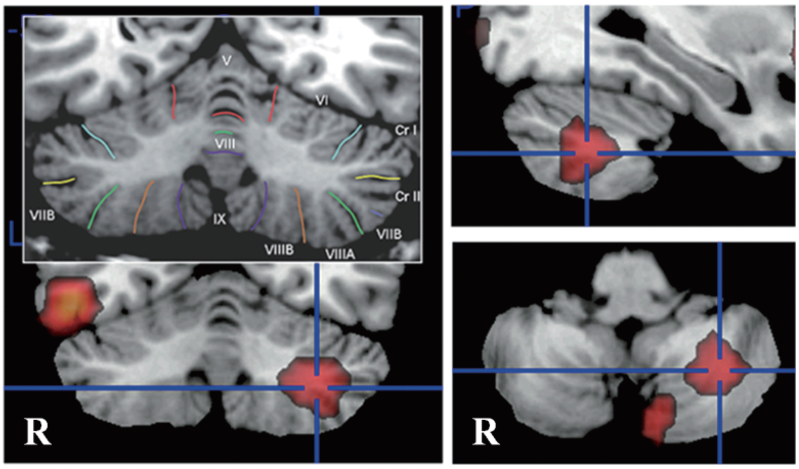

Fig. 2. Localisation of the Crus II cluster on the standard MNI template. Inset: comparable section with labels taken from atlas of Schmahmann et al. (2000). (Radiological orientation).

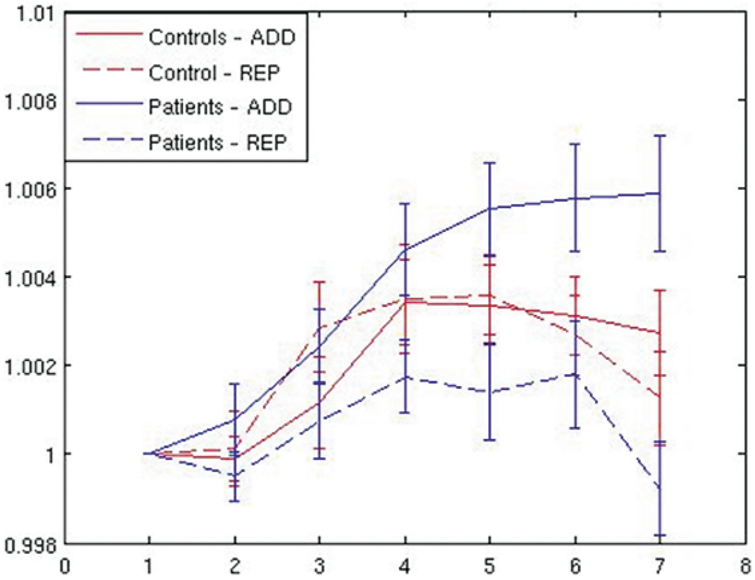

Fig. 3. Mean peri-stimulus time plot of BOLD activity of Crus II cluster. Signal change relative to 1TR before block onset.

bral pathology. Our results are in line with theoretical models of information processing and plasticity in the cortico-cerebellar system, and with empirical evidence in healthy humans that implicates this area of the cerebellar cortex in the acquisition and execution of cognitive skills [3,44,54,66,68].

Our approach differs from other studies in important ways. First, we employed a variant of the PASAT that remained faithful to the original form during fMRI, where the auditory and verbal task demands were preserved [46]. Second, it is important to apply analytical techniques such as Independent Component Analysis (ICA) that are as free from assumptions as possible, particularly in patient populations in which neurovascular coupling and the form of the hemodynamic response to a given stimulus may differ from the models that assume a form that is present in healthy populations [52,80], thus minimizing the risk of Type I errors (i.e. failure to detect activations when they are present). We are the first to apply a combined model-free (ICA) and model-based (GLM) approach in a population of MS patients. Third, these methodological approaches allowed us to relate the process of compensation to theoretical models of learning-related plasticity and information processing in the cortico-cerebellar system [57, 77].

An important concept in studies of systems-level reorganisation following central nervous system pathology is the idea that brain systems contain inherent redundancy, sometimes referred to as ‘degeneracy’ [73]. That is, performance of cognitive or motor tasks can be achieved using several alternative pathways, and lesions in one particular pathway may result in alternative pathways being used to perform with similar levels of competence. Functional imaging can unmask these alternative pathways by scanning patients who perform similarly to control counterparts despite the presence of pathology [72]. Additionally, it is important to demonstrate that activity in such pathways is explained by the presence of a condition-specific difference that differs between groups. Here, we have shown that cerebellar cortical areas that normally support the execution of the PASAT in the healthy brain show interactions, where the main effect of Group and the main effect of Condition by themselves are insufficient to explain the activity. It is the confluence of these factors that explains the activity, and suggests a special role for this area in supporting cognitive skills related to the PASAT in RRMS patients.

We have also argued that these systems are likely to be the ones that are already well equipped for supporting the process of learning, and our evidence supports the view that the cortico-cerebellar system is no exception. The organization and physiology of this system offers clues about its nature of its plasticity and information flow. Prominent models of information processing in this system suggest that cerebellar cortical plasticity enables the acquisition and storage of models of cortical information processing [3,44,54,66,68, 91]. There is empirical evidence to support the view that the cerebellar cortex stores internal models used for motor learning [83], the predictive processing of sensory information [53] and the use of symbolic cues to guide action [12]. The deployment of these models enables the efficient and relatively automatic execution of information processing that is robust to the effects of distracters, such as concurrently performed tasks. It has been suggested for some time that there is a functional topography in the cerebellar cortex in which areas of the prefrontal cortex and the cortical motor areas project to distinct zones and are likely to perform the same computational operations to these diverse inputs. In particular, cerebellar lobule HVII is known to have dense connections with prefrontal area 46 [56], and we have suggested that it is specifically this area that is likely to be engaged in the acquisition of cognitive skills ([12,77],subsequently, Stoodley and Schmahmann [86] elegantly demonstrated the generality of this finding in a meta-analysis). Desmond and colleagues [30,32] have reported the involvement of the same areas of the cerebellar cortex and prefrontal cortex during verbal working memory. They go further to fractionate these areas into components that contribute to articulatory control and phonological storage [26,27, 33].

It is important to consider how this anatomical system works in the presence of pathology. Lesions to lobule VII (including Crus II), have been associated with deficits generally observed after frontal-lobe damage [37,82]. However, can skills that are known to be cerebellar-dependent still operate in the absence of normal function in the forebrain? Work in animals shows that removal of the cerebral cortex, or even the entire forebrain, abolishes behavioural manifestations of skills such as the conditioning of motor responses. In theory, the execution of skills (whether motor or cognitive) might therefore be supported by cerebellar circuitry in the face of failures of cortical information processing. In line with the idea of cerebellar regions compensating for generalized CNS damage, Desmond et al. [31] found increased cerebellar lobule VII and prefrontal activity in alcoholics compared to controls in a verbal working memory task. We suggest that cerebellar Crus II might play an important role in maintaining skilled cognitive performance in RRMS during remission in the face of diffuse and advancing pathology in the cerebral cortex.

It is also important to relate compensation to systems level theories that explain the ways that areas of the cortex communicate with each other and the ways in which this information processing is supported by the cerebellum. The PASAT requires the coordination of several cognitive processes in the healthy brain, and is likely supported by the interactions between several cortical areas [50]. White matter loss that results in delayed transmission of information between areas would inevitably result in increasingly inefficient information processing. Control theoretic accounts suggest that in the healthy brain the circuits of the cerebellar cortex acquire internal models of cortical information processing. During cognitive tasks, these cerebellar representations enable the rapid and automatic instantiation of cognitive operations with minimal interaction with the prefrontal cortex. We suggest that in RRMS patients in whom such models have been acquired (our patients were pretrained on the task before scanning), these cerebellar operations may substitute for particular failures of information processing in the prefrontal cortex that may come about because of impaired cortico-cortical connectivity. At a clinical level, if the role of the cerebellum in cognition is to automate repetitive, skilled tasks, then the vulnerability of the cerebellum to MS pathology may also impact on cognitive impairment and other disease outcomes [61, 81]. Whilst our RRMS patient group were in remission and therefore had no physical cerebellar symptoms of note, reports of patients in later stages of the disease with marked cerebellar symptoms suggest that clinical cerebellar dysfunction is associated with poor prognosis in terms of disability and physical rehabilitation benefit [5,62]. Cerebellar pathology may result in reduced automation to support motor and cognitive processes. This may impose an additional slowing of complex processing, which characterizes cognitive impairment in MS, above that occurring as a primary result of demyelination of the white matter tracts and inefficient data transfer [43,88].

## Acknowledgements

ALH and MRI data collection were supported by an unrestricted educational grant from Bayer Healthcare to DWL. NR supported by a grant from BBSRC.
